# Semaglutide use for hypothalamic obesity and type 2 diabetes mellitus after suprasellar germinoma treatment

**DOI:** 10.1210/jcemcr/luag107

**Published:** 2026-05-18

**Authors:** Kenichi Yokota, Tomoko Nakagawa, Yuta Nakamura, Nanami Okada, Shuhei Kusuda, Masakatsu Sone

**Affiliations:** Division of Metabolism and Endocrinology, St. Marianna University School of Medicine, Kawasaki 216-8511, Japan; Division of Metabolism and Endocrinology, St. Marianna University School of Medicine, Kawasaki 216-8511, Japan; Division of Metabolism and Endocrinology, St. Marianna University School of Medicine, Kawasaki 216-8511, Japan; Division of Metabolism and Endocrinology, St. Marianna University School of Medicine, Kawasaki 216-8511, Japan; Division of Metabolism and Endocrinology, St. Marianna University School of Medicine, Kawasaki 216-8511, Japan; Division of Metabolism and Endocrinology, St. Marianna University School of Medicine, Kawasaki 216-8511, Japan

**Keywords:** hypothalamic obesity, diabetes mellitus, GLP-1 receptor agonists, semaglutide

## Abstract

Hypothalamic obesity (HO) is a severe complication of suprasellar tumors characterized by hyperphagia, rapid weight gain, and resistance to lifestyle interventions. We describe a case of a woman diagnosed with suprasellar germinoma at 16 years of age who was treated with chemotherapy and radiotherapy. Subsequently, she experienced progressive weight gain, increasing from 57 kg to a peak of 138 kg by age 37 years, and developed type 2 diabetes mellitus at age 28 years. At age 38 years, she presented with polydipsia, polyuria, and fatigue, and was admitted for the management of hyperglycemia with class III obesity (123.5 kg; body mass index [BMI], 51.4 kg/m^2^). Intensive insulin therapy (up to 100 units/day) resolved glucotoxicity. After initiation of semaglutide (titrated from 0.25 to 0.5 mg/week), appetite markedly decreased, and subsequent weight loss was achieved. Three months after discharge, her weight decreased from 131 kg to 112.1 kg, representing an approximate 14% reduction, accompanied by optimized glycemic control. Body composition analysis revealed that weight loss was primarily due to fat mass reduction with relative preservation of muscle mass. This case demonstrates the potential efficacy of semaglutide in patients with HO and type 2 diabetes mellitus after suprasellar germinoma treatment.

## Introduction

Hypothalamic obesity (HO) is a severe metabolic complication frequently observed in survivors of suprasellar tumors, such as craniopharyngiomas and germinomas, primarily resulting from surgical resection or radiotherapy involving the hypothalamic region [1]. The pathophysiology of HO involves structural damage to key appetite-regulating nuclei, which disrupts signaling pathways that regulate satiety and energy expenditure [1, 2]. Consequently, patients experience intractable hyperphagia, rapid weight gain, and severe metabolic comorbidities [3]. Crucially, HO is resistant to conventional lifestyle interventions, such as caloric restriction and increased physical activity [1, 3].

Recently, glucagon-like peptide-1 receptor agonists (GLP-1 RAs) have emerged as promising therapeutic agents for HO. Although physiological satiety signals typically rely on intact hypothalamic circuits, GLP-1 RAs may exert anorexigenic effects through alternative pathways independent of the hypothalamus, such as vagal afferents and the hindbrain [4, 5]. A randomized clinical trial demonstrated the efficacy of exenatide in reducing body mass index (BMI) in young patients with HO [6]. More recently, real-world studies and case series have reported significant weight loss with semaglutide treatment in adults with HO, suggesting potential superiority and broader applicability [7-11].

However, most existing literature on HO has focused on patients after craniopharyngioma treatment, and reports on HO secondary to suprasellar germinoma remain scarce. Furthermore, the specific effect of semaglutide on body composition in the context of HO remains to be elucidated. We report a case of a woman with Class III obesity and type 2 diabetes mellitus secondary to suprasellar germinoma.

## Case presentation

A 38-year-old Japanese woman with Class III obesity presented with polydipsia, polyuria, and fatigue, and was admitted for management of hyperglycemia. Her medical history included suprasellar germinoma diagnosed at age 16 years and treated with chemotherapy (cisplatin and etoposide) and radiotherapy (total dose, 54.2 Gy). As a result, she developed permanent panhypopituitarism, managed with hydrocortisone (20 mg/day in three divided doses: 10 mg in the morning, 5 mg in the afternoon, and 5 mg in the evening), levothyroxine (125 mcg daily), conjugated estrogens (0.625 mg twice daily), medroxyprogesterone (2.5 mg twice daily), and oral desmopressin (120 mcg every 8 hours).

After tumor treatment, she developed marked hyperphagia and a significant loss of satiety. As a result, her weight increased progressively from 57 kg (BMI, 23.7 kg/m^2^) at age 16 to a peak of 138 kg (BMI, 57.4 kg/m^2^) at age 37. In addition, she had developed type 2 diabetes mellitus at age 28 years. Despite treatment with oral antidiabetic agents (metformin and vildagliptin) and previous hospitalizations for weight management, her condition deteriorated because of rebound weight gain and poor adherence.

## Diagnostic assessment

On admission, physical examination revealed Class III obesity (height, 155 cm, weight, 123.5 kg; BMI, 51.4 kg/m^2^). Vital signs were unremarkable except for hypertension (145/89 mmHg). In addition to oral antidiabetic agents (metformin, 2000mg/day and vildagliptin, 100 mg/day), she was taking pitavastatin (1 mg daily) for dyslipidemia and cilostazol (100 mg twice daily) and limaprost alfadex, a prostaglandin E1 derivative (5 mg three times daily), for peripheral artery disease (PAD). She had never previously been treated with any weight-reducing agents, including SGLT2 inhibitors, GLP-1 RAs, or dual GIP (glucose-dependent insulinotropic polypeptide)/GLP-1 RAs. She was a nonsmoker and an occasional social drinker. Laboratory findings showed severe hyperglycemia (random plasma glucose, 412 mg/dL [SI: 22.9 mmol/L] [reference range, 73–109 mg/dL {SI: 4.0-6.0 mmol/L}]; hemoglobin A1c [HbA1c], 12.6% [SI: 114 mmol/mol] [reference range, 4.9-6.0% {SI: 30-40 mmol/mol}]) with preserved endogenous insulin secretion (serum C-peptide 4.0 ng/mL [SI: 1.32 nmol/L] [reference range, 0.61-2.09 ng/mL {SI: 0.20-0.69 nmol/L}]) when the fasting glucose level was 241 mg/dL [SI: 13.4 mmol/L] and urinary C-peptide was 93.6 µg/day [SI: 31.0 nmol/day] [reference range, 41.0-169.0 µg/day {SI: 13.6-56.0 nmol/day}]) (Table 1). Evaluation of diabetic microvascular complications revealed neither diabetic retinopathy nor diabetic polyneuropathy. Nephropathy assessment showed normal renal function with a urinary albumin-to-creatinine ratio of 5.9 mg/day (reference range, <30.0 mg/day) (chronic kidney disease [CKD] stage G1A1). Evaluation of macrovascular complications revealed decreased blood flow in the left lower limb, as indicated by the ankle-brachial index (ABI) and confirmed by lower-extremity magnetic resonance imaging (MRI). Pituitary function tests confirmed panhypopituitarism. MRI showed no germinoma recurrence.

**Table 1:** Laboratory findings on admission

Parameter	Value	Reference values
**Hematology**		
White blood cell count	8.30 × 10^3^/µL (8.30 × 10^9^/µL)	3.3-8.6 × 10^3^/µL (3.3-8.6 × 10^9^/µL)
Hemoglobin	11.8 g/dL (118 g/L)	11.6-14.8 g/dL (116-148 g/L)
Platelet count	32.8 × 10^4^/µL (328 × 10^9^/L)	15.8-34.8 × 10^4^/µL (158-348 × 10^9^/L)
**Biochemistry**		
Total protein	6.6 g/dL (66 g/L)	6.6-8.1 g/dL (66-81 g/L)
Albumin	3.5 g/dL (35 g/L)	4.1-5.1 g/dL (41-51 g/L)
Aspartate transaminase	28 U/L (28 U/L)	13-30 U/L (13-30 U/L)
Alanine aminotransferase	35 U/L (35 U/L)	7-23 U/L (7-23 U/L)
Alkaline phosphatase	103 U/L (103 U/L)	38-113 U/L (38-113 U/L)
γ-Glutamyl transferase	47 U/L (47 U/L)	9-32 U/L (9-32 U/L)
Creatine kinase	112 U/L (112 U/L)	41-153 U/L (41-153 U/L)
Amylase	54 U/L (54 U/L)	44-132 U/L (44-132 U/L)
Urea nitrogen	9.8 mg/dL (3.5 mmol/L)	8.0-20.0 mg/dL (2.9-7.1 mmol/L)
Creatinine	0.55 mg/dL (48.6 µmol/L)	0.46-0.79 mg/dL (40.7-69.8 µmol/L)
Uric acid	5.8 mg/dL (345 µmol/L)	2.6-5.5 mg/dL (154.6-327.1 µmol/L)
Sodium	136 mEq/L (136 mmol/L)	138-145 mEq/L (138-145 mmol/L)
Potassium	4.1 mEq/L (4.1 mmol/L)	3.6-4.8 mEq/L (3.6-4.8 mmol/L)
Chloride	102 mEq/L (102 mmol/L)	101-108 mEq/L (101-108 mmol/L)
**Glucose and lipids**		
Triglycerides	174 mg/dL (1.97 mmol/L)	30-149 mg/dL (0.34-1.68 mmol/L)
HDL-cholesterol	35 mg/dL (0.91 mmol/L)	40-119 mg/dL (1.03-3.08 mmol/L)
LDL-cholesterol	110 mg/dL (2.84 mmol/L)	70-139 mg/dL (1.81-3.60 mmol/L)
Random plasma glucose	412 mg/dL (22.9 mmol/L)	73-109 mg/dL (4.0-6.0 mmol/L)
Hemoglobin A1c	12.6% (114 mmol/mol)	4.9-6.0% (30-42 mmol/mol)
Glycated albumin	23.0% (23.0%)	11.6-16.4% (11.6-16.4%)
C-peptide	4.0 ng/mL (1.32 nmol/L)*^a^*	0.61-2.09 ng/mL (0.20-0.69 nmol/L)
**Endocrinology** *^b^*		
TSH	0.04 µU/mL (0.04 mIU/L)	0.61-4.23 µU/mL (0.61-4.23 mIU/L)
Free T3	2.01 pg/mL (3.10 pmol/L)	2.39-4.06 pg/mL (3.68-6.24 pmol/L)
Free T4	0.80 ng/dL (10.3 pmol/L)	0.76-1.65 ng/dL (9.8-21.2 pmol/L)
ACTH	<1.5 pg/mL (<0.33 pmol/L)	7.2-63.3 pg/mL (1.58-13.9 pmol/L)
Cortisol	15.0 µg/dL (413.9 nmol/L)	7.1-19.6 µg/dL (195.9-540.8 nmol/L)
DHEA-S	<2 µg/dL (<0.05 µmol/L)	106-448 µg/dL (2.88-12.16 µmol/L)
Growth hormone	<0.03 ng/mL (<0.03 µg/L)	0.13-9.88 ng/mL (0.13-9.88 µg/L)
IGF-1	19 ng/mL (2.49 nmol/L)	121-236 ng/mL (15.9-30.9 nmol/L)
Prolactin	6.23 ng/mL (6.23 µg/L)	4.91-23.32 ng/mL (4.91-23.32 µg/L)
**Urinalysis**		
Urinary glucose	4+	Negative
Urinary albumin	5.9 mg/day (5.9 mg/day)	<30.0 mg/day (<30.0 mg/day)
Urinary C-peptide	93.6 µg/day (31.0 nmol/day)	41.0-169.0 µg/day (13.6-56.0 nmol/day)
**Tumor Markers & Immunology**		
CEA	3.9 ng/mL (3.9 µg/L)	<5.0 ng/mL (<5.0 µg/L)
CA19-9	10.6 U/mL (10.6 kU/L)	<37.0 U/mL (<37.0 kU/L)
Anti-GAD antibody	<5.0 U/mL (<5.0 U/mL)	<5.0 U/mL (<5.0 U/mL)

The values in parentheses are the International System of Units (SI).

Abbreviations: ACTH, adrenocorticotropic hormone; CA19-9, carbohydrate antigen 19-9; CEA, carcinoembryonic antigen; DHEA-S, dehydroepiandrosterone sulfate; GAD, glutamic acid decarboxylase; GH, growth hormone; HDL, high-density lipoprotein; IGF-1, insulin-like growth factor-1; LDL, low-density lipoprotein; T3, triiodothyronine; T4, thyroxine; TSH, thyroid-stimulating hormone.

^*a*^Measured when the fasting plasma glucose level was 241 mg/dL.

^*b*^Measured under hormone replacement therapy (hydrocortisone, levothyroxine, conjugated estrogens, and medroxyprogesterone acetate).

## Treatment

During hospitalization, she was placed on a 1400 kcal/day therapeutic diet; nevertheless, repeated episodes of secretive consumption of soft drinks and snacks were noted, and improvement remained difficult despite repeated counseling. Her recent daily dietary habits and estimated energy intake are summarized in Table 2.

**Table 2:** Daily dietary habits and estimated energy intake at presentation

Category	Estimated energy intake (kcal/day)	Content
Breakfast	550∼650	Two slices of toasted bread with honey and margarine, ham, lettuce Tea/coffee with 1 tablespoon of sugar (15 g) or fruit juice yogurt
Lunch	450∼600	Ready-to-eat pasta or instant noodles
Snack (3:00 Pm)	500∼700	Tea/coffee with 1 tablespoon of sugar (15 g) 100% fruit juice, or cola Ice cream, rice crackers, chocolate, or savory snacks
Dinner	850∼1000	Steamed rice (180 g), miso soup, meat or fish, deep-fried side dishes, tomato, salad
Fruit	50	1/2 apple or assorted cut fruits (consumed in the morning or evening)
Beverages	450	Sugar-sweetened beverages (fruit juice or carbonated drinks, eg, cola): approximately 1 L/day
Total	2850∼3450	—
Others	—	Episodic binge eating several times per month

**Figure 1:**
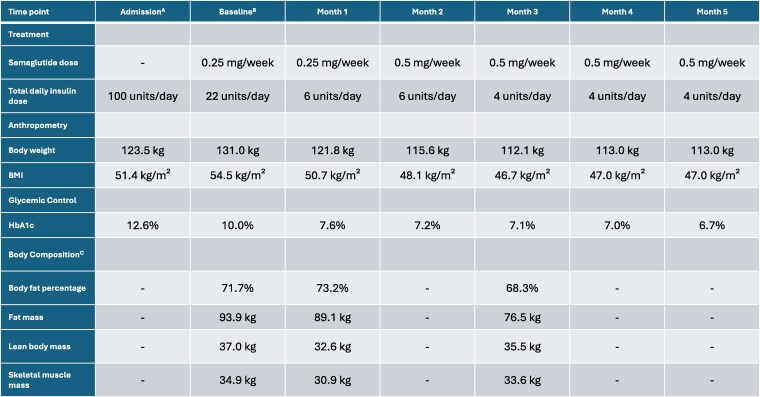
Clinical course of body weight, glycemic control, and body composition. Values for body composition were not measured at Admission, Month 2, 4, and 5. A Data at the first admission for severe hyperglycemia. B Data at the initiation of semaglutide treatment (0 months). C Body composition was measured using an MC-180 Body Composition Analyzer (Tanita Corp., Tokyo, Japan) according to the manufacturer's instructions. Abbreviations: BMI, body mass index; HbA1c, hemoglobin A1c.

Intensive insulin therapy was initiated, and insulin requirements subsequently escalated to 100 units/day (insulin lispro, 26–28–20 units/day; insulin glargine, 26 units/day; total, 0.76 units/kg/day) to achieve glycemic stability. However, during this period, her weight further increased to 131 kg (BMI, 54.5 kg/m^2^) (Fig. 1). Subsequently, semaglutide (0.25 mg subcutaneously once weekly) was added. Remarkably, the patient reported a decrease in appetite, and food intake decreased spontaneously without significant adverse events, such as severe vomiting, diarrhea, or constipation. She was discharged on semaglutide (0.25 mg/week), metformin (2000 mg/day), and insulin glargine (22 units/day).

## Outcome and follow-up

One month after discharge, semaglutide was increased to 0.5 mg/week, and insulin was tapered to 6 units/day as glycemic control improved, which consequently supported sustained weight management. After this modification, the patient demonstrated further weight loss and improved glycemic control. Three months after discharge, her body weight decreased to 112.1 kg (−18.9 kg) and her BMI to 46.7 kg/m^2^ (−7.8 kg/m^2^), representing an approximate 14% reduction from baseline (Fig. 1). Body composition analysis using a body composition analyzer (MC-180; Tanita Corp., Tokyo, Japan) revealed that fat mass reduction (17.4 kg) accounted for approximately 92% of total weight loss, with relative preservation of lean muscle mass (Fig. 1). By 5 months, HbA1c improved to 6.7% (SI: 50 mmol/mol) using only 4 units/day of basal insulin and metformin (2000 mg/day).

## Discussion

This case highlights the efficacy of semaglutide in the treatment of intractable HO complicated by severe type 2 diabetes mellitus. The patient experienced substantial weight loss and achieved optimal glycemic control with minimal insulin requirements. Our case offers three novel clinical insights [1]: the remarkable efficacy of a relatively low dose (0.25-0.5 mg/week) of semaglutide [2], a favorable weight loss profile characterized by fat mass reduction with relative preservation of lean mass, and [3] successful disruption of the vicious cycle of high-dose insulin and weight gain.

HO typically results from damage to the ventromedial hypothalamus (VMH), which disrupts satiety signaling [1, 2]. While GLP-1 RAs act on the hypothalamic nuclei, they also exert anorexigenic effects via the hindbrain (eg, area postrema and nucleus of the solitary tract) and vagal afferents [3, 4]. Recent real-world studies, including a large series from China by Wang et al [11]. and European cohorts [7, 8], have confirmed that semaglutide is effective for HO.

A striking feature of our case is responsiveness to a relatively low therapeutic dose (0.25-0.5 mg/week), which resulted in a weight reduction from 131 to 113 kg (BMI, from 54.5 kg/m^2^ to 47.0 kg/m^2^), and an improvement in HbA1c from 12.6% (SI: 114 mmol/mol) to 6.7% (SI: 50 mmol/mol) at 5 months after discharge. A previous study from Denmark [7] reported that a median dosage of semaglutide of 1.6 mg/week achieved a mean weight loss of 13.4 kg (−4.4 kg/m^2^ in BMI) at 12 months. Wang et al [11]. reported a mean weight loss of 12.8 kg (−4.0 kg/m^2^ in BMI) at 6 months in Chinese patients receiving a relatively low dose of semaglutide (0.67 mg/week). These findings suggest that responsiveness to semaglutide in HO may vary by ethnicity. Our patient achieved an 18.9 kg weight loss (−7.8 kg/m^2^ in BMI) with 0.25-0.5 mg/week at 3 months—significantly lower than the conventional dose used for HO treatment—suggesting that she may be a “hyper-responder” to GLP-1 RAs. This high sensitivity may be partially explained by the underlying etiology. While most previous studies predominantly focused on HO caused by craniopharyngioma [7-11], our patient survived germinoma. Differences in the nature of hypothalamic injury, which often vary between extensive surgical resection in craniopharyngioma and chemoradiotherapy in germinoma, might influence responsiveness to GLP-1 RAs. Consequently, low-dose strategies may be sufficient for a subset of patients with HO, thereby improving cost-effectiveness and tolerability.

Furthermore, the quality of weight loss in our patient was favorable. Rapid weight loss often leads to sarcopenia, which is particularly concerning in patients with panhypopituitarism who lack growth hormone and gonadal steroids. However, body composition analysis revealed that 92% of weight loss was attributable to fat mass, with minimal reduction in lean body mass. Although Gjersdal et al [8]. reported improvements in body composition in patients with HO after craniopharyngioma treatment, detailed data on HO after suprasellar germinoma treatment remain scarce. We hypothesize that resolution of severe insulin resistance and glucotoxicity, facilitated by semaglutide and insulin therapy, restores anabolic insulin signaling in skeletal muscle, thereby preventing catabolism despite negative energy balance.

Semaglutide successfully disrupts the vicious cycle of insulin-induced weight gain. Prior to initiation, the patient required 100 units/day of insulin, a regimen that paradoxically exacerbates weight gain. Semaglutide not only suppressed appetite but also improved insulin sensitivity to such an extent that the total daily insulin dose could be reduced to 4 units/day. This dramatic reduction in insulin requirements distinguishes our case from others and underscores the value of semaglutide as a first-line treatment for patients with HO.

This study had several limitations. First, as a single-case study, the generalizability of our findings is limited. HO is a heterogeneous condition with varying degrees of neuronal damage. Therapeutic responses may differ among individuals depending on the extent of hypothalamic preservation. Second, in this case, thyroid hormone levels remained in the low-normal range for a long period before admission. Such suboptimal management may have exacerbated her weight gain alongside hypothalamic injury. Third, the observation period was relatively short; therefore, the long-term sustainability of weight loss, glycemic control, and safety requires further investigation. Large-scale clinical trials are warranted to establish the efficacy and optimal treatment protocols of semaglutide in this population.

In conclusion, low-dose semaglutide provides comprehensive metabolic benefit for patients with HO and type 2 diabetes mellitus. Future studies should investigate predictors of high sensitivity to GLP-1 RAs to optimize personalized treatment strategies for this challenging condition.

## Learning points

- Semaglutide represents a promising therapeutic option for intractable HO after suprasellar germinoma treatment with coexisting type 2 diabetes mellitus.
- Weight reduction achieved with semaglutide in patients with HO is primarily attributable to a significant decrease in fat mass with relative preservation of lean body mass, resulting in favorable metabolic outcomes.
- Semaglutide may effectively disrupt the “vicious cycle” of high-dose insulin requirements and subsequent weight gain in patients with Class III obesity and severe insulin resistance secondary to HO.

## Contributors

All authors made individual contributions to the authorship. K.Y., T.N., Y.N., and M.S. were involved in the diagnosis and management of this patient. N.O. and S.K. were involved in the acquisition and interpretation of clinical and body composition data. K.Y., T.N., Y.N., and M.S. performed the literature search and drafted the manuscript. All authors reviewed and approved the final draft.

## Data Availability

Original data generated and analyzed during this study are included in this published article.
